# The role of the oncofetal H19 lncRNA in tumor metastasis: orchestrating the EMT-MET decision

**DOI:** 10.18632/oncotarget.6387

**Published:** 2015-11-25

**Authors:** Imad J. Matouk, David Halle, Eli Raveh, Michal Gilon, Vladimir Sorin, Avraham Hochberg

**Affiliations:** ^1^ Department of Biological Chemistry, Institute of Life Sciences, The Hebrew University of Jerusalem, Jerusalem, Israel; ^2^ Department of Biological Sciences, Faculty of Science and Technology, Al-Quds University, Jerusalem, West Bank

**Keywords:** H19, miR-675, metastasis, lncRNA, EMT, MET

## Abstract

Long non-coding RNA (lncRNA) genes are emerging as key players in the metastatic cascade. Current evidence indicate that H19 lncRNA and the microRNA(miRNA) miR-675, which is processed from it, play crucial roles in metastasis, through the regulation of critical events specifically the epithelial to mesenchymal (EMT) and the mesenchymal to epithelial transitions (MET). This review summarizes recent mechanistic pathways and tries to put together seemingly conflicting data from different reports under one proposed general scheme underlying the various roles of H19/miR-675 in the metastatic cascade. We propose several approaches to harnessing this knowledge for translational medicine.

## INTRODUCTION

Approximately 90% of cancer related deaths can be attributed to metastasis. Metastasis is a complex multistep process, through which a minority of well adapted tumor cells manages to leave the primary site and settle in distant organ to generate new foci of growth, causing failure of essential organs including the lungs, liver, brain and bone marrow. The metastatic journey can be accomplished via various routes through the circulatory system and/or through the lymphatic system. Metastatic spread is regarded as a highly inefficient process and the trip can take a long time, even several years. Recent evidence indicates that at least for some metastatic tumor types, it begins very early in tumorigenesis, contrary to what was believed earlier [1].

Primary tumor microenvironment interactions with cancer cells are pivotal determinants of the metastatic potential. There is a dynamic state of crosstalk between primary tumor cells and components of the microenvironment that dictate important transitions towards the metastatic phenotype. For example, cytokines secreted by cancer cells can activate stromal fibroblasts and recruit immune cells to the tumor primary location that pave the way towards a remodeled tumor microenvironment supportive of invasion [2]. In addition, the EMT process that tumor cells undergo to enable their motility and invasion and subsequent dissemination, can largely be triggered by secreted growth factors and cytokines contributed by stromal cells [3]. Other types of interactions in the blood stream also seem essential determinants of the metastatic potential. For example, during intravascular transit, tumor cells receive instructive signals while in the circulation or upon arrival at the secondary site that greatly influence metastatic ability. It was shown for instance that circulating tumor cells interact directly with platelets which secrete cytokines such as transforming growth factor beta (TGF-β) to activate TGF-β/Smad and NF-κB pathways in cancer cells thus promoting the metastatic phenotype [4]. Furthermore, platelets can form a physical shield around circulating tumor cells protecting them from components of the immune system, particularly natural killer cells, limiting their exposure to shear stress, and promoting their adhesion to the endothelium.

Recent data show that cancer progression cannot be attributed to the underlying genetic alterations of cancer cells and the paracrine interactions within the tumor microenvironment alone, but also may be directed by the body's systemic responses to malignancy and the involvement of organ systems at the secondary growth sites (for review see [5]). Systemic responses triggered by pre-metastatic primary tumors can dictate the site of extravasation of subsequently disseminated cancer cells. This can be accomplished by the secretion of a variety of cytokines, chemokines and growth factors that can remodel the microenvironment of the secondary metastatic site to be more competent niche for colonization and establishment of new foci of growth [6, 7]. Pre-metastatic niche formation can also be accomplished via exosomes (small membrane vesicles of 30–100 nm diameter) generated by primary tumors that can mediate both local and systemic cell communication through transferring mRNAs, miRNAs and proteins to the recipient cells, thus altering their phenotype. This was reported for example in melanoma derived exosomes which can induce vascular leakiness at pre-metastatic sites and reprogram bone marrow progenitors toward a pro-vasculogenic phenotype [8].

Understanding the molecular mechanisms governing tumor metastatisis in all its facets is vitally important. Despite the very long time that has passed since the process was described for the first time and the wealth of information about the biology of this process, very little has been done to exploit this knowledge for the benefit of cancer patients.

## LONG NON-CODING RNA'S DIVERSE ROLES IN METASTASIS - AN OVERVIEW

Our genome is regarded as a transcription machine with the vast majority (∼98%) of its transcriptional output being non-coding RNA. Non-coding RNA falls into various categories with RNA of more than 200 bases in length termed long non-coding RNA (lncRNA). Essential roles for many lncRNAs in diverse physiological cellular processes have been discovered in recent years and deregulated expression has been linked to many diseases including cancer, whereby the lncRNA functions either as a tumor suppressor or as an oncogene involved in tumor initiation and progression.

Table 1 lists several lncRNA genes with established roles in the metastatic process. This table does not comprehensively list all the lncRNA genes functioning in metastasis, but rather genes have been selected in order to illustrate the diverse mechanisms through which they can perform their pro-metastatic or anti-metastatic functions [9-32].

In contrast to miRNAs, which act mainly as post-transcriptional repressors, lncRNAs in addition can perform their functions through diverse mechanisms. These molecules act as decoys, guides, or scaffolds for their interacting proteins, such as transcription factors and histone modifiers. LncRNA can interact with chromatin remodeling machinery leading to changes in chromatin packaging. This mode of action is a common theme for many lncRNAs. Some examples are listed in table 1. Perhaps the most well established example is reported for HOTAIR [11]. HOTAIR forced expression induced genome-wide retargeting of Polycomb repressive complex 2 (PRC2) leading to altered histone H3 lysine 27 methylation and increased cancer invasiveness and metastasis [11]. Another example is reported for ANRIL (CDKN2B-AS1), a 3.8-kb lncRNA could epigenetically silence miR-99a/miR-449a by binding to PRC2, thus regulating mTOR and CDK6/E2F1 pathway [21]. The concept of epigenomic reprogramming by lncRNAs in the context of metastasis is also applicable to so many other examples (Table 1).

**Table 1:** Selected metastasis-related lncRNAs showing different and diverse modes of action

Gene symbol	Mechanism of action.	References
MALAT1	Interacts with Ezh2 resulting in suppression of E-cadherin and activation of β-catenin.	[9].
HOTAIR	Competing endogenous RNA. Sponges miR-331-3p to regulate HER2 expression. Reprograms chromatin state.	[10,11].
HNF1A-AS1	Binds DNMT1 to regulate E-cadherin.	[12].
H19	Precursor miR-675. Sponger of Let-7. Chromatin modifier.	[13-15].
HULC	Liver metastasis specific lncRNA. Affected by liver micro-environment.	[16].
lncRNA-ATB	Up-regulates ZEB1 and ZEB2 by competitively binding the miR-200 family, inducing EMT.	[17].
BANCR	Histone de-acetylation suppresses BANCR to promote EMT.	[18].
SPRY4-IT1	Epigenetically suppressed by Ezh2, to promote EMT.	[19].
ANRIL	Crosstalk with microRNAs at epigenetic level	[20,21].
UCA1	Endogenous sponge for miR-216b, relieving its inhibitory effect on metastasis promoting genes.	[22].
NKILA	Interacts with NF-κB/ IκB to form a stable complex. Directly blocks IκB phosphorylation.	[23].
lncRNA-HIT	Mediates TGFβ function, regulates EMT, invasion and metastasis.	[24].
SChLAP1	Antagonizes the genome-wide localization and regulatory functions of the SWI/SNF chromatin-modifying complex.	[25].
lncRNA-LET	Suppressed by hypoxia through histone de-acetylation resulting in stabilization of nuclear factor 90 protein, and so metastasis.	[26].
Zeb2-NAT.	Natural antisense transcript. Prevents splicing of the Zeb2 5′-UTR, increases Zeb2 and down-regulates E-cadherin.	[27].
91H	H19 antisense. Associated with H19 ICR methylation. Inhibits IGF2 expression	[28,29].
HOTTIP/HOXA13	Bidirectional regulatory loop involved in metastasis and survival of HCC.	[30].
linc-UBC1	Physically associates with the PRC2 complex.	[31].
ZEB1-AS1	Upstream antisense RNA enhances ZEB1 expression.	[32]

LncRNAs can also perform their functions by serving as precursors and/or as spongers for miRNAs. H19 lncRNA for example can perform both of these modes of action and will be discussed below [13, 14]. Competing for endogeneous miRNA is a widespread scenario and have been recently reported for many other lncRNAs. The urothelial cancer associated 1 (UCA1) lncRNA for example can serve as an endogenous sponge for miR-216 b, which might be involved in the derepression of fibroblast growth factor receptor 1 (FGFR1) expression, a target gene of miR-216b, and the activation of extracellular-signal-regulated kinase (ERK) signaling pathway [22]. Furthermore, it was recently reported that HOTAIR upregulation is a characteristic molecular change in gastric cancer. HOTAIR regulate the expression of human epithelial growth factor receptor 2 (HER2) through sponging miR-331-3p, [10]. Moreover a lncRNA-activated by TGF-β (lncRNA-ATB) was reported recently to upregulate ZEB1 and ZEB2 by competitively binding the miR-200 family and then induced EMT and invasion [17].

Furthermore, emerging studies showed that several lncRNA can influence metastasis by serving as antisense transcripts sometimes emanating from the promoters of corresponding genes. Recent studies showed that upstream antisense transcription may function as an enhancer for corresponding gene expression. For example it was recently reported that ZEB1-AS1 can induce EMT by up regulating ZEB1 expression, through increasing its promoter activity [32].

The existence of an antisense transcript was also reported for Zeb-2. Ectopic over-expression of this antisense transcript in epithelial cells prevents splicing of the *Zeb2* 5′-UTR, increases the levels of Zeb2 protein, and consequently down-regulates E-cadherin mRNA and protein. [27].

Further examples of lncRNA serving as antisense transcripts and influencing metastasis include 91H [28, 29], ARNL( CDKN2B-AS1) [20, 21], and HNF1A-AS1 [12].

## MASTER REGULATORS OF EMT INDUCE H19 GENE EXPRESSION

In epithelial cancer, acquisition of invasiveness is often accompanied by a loss of epithelial features and a gain of mesenchymal features, a process known as epithelial to mesenchymal transition (EMT). A growing list of molecular and environmental cues can initiate, maintain or revert EMT.

Several signaling pathways have relatively well established roles in the induction of EMT through the so-called EMT transcription factors. These pathways include those mediated by TGF-β; by hepatocyte growth factor, also known as scatter factor, (HGF/SF) via MET receptor; the Wnt/β-catenin signaling pathway; and signaling pathways that mediate the multi-drug resistance phenotype. There is a growing list of EMT transcription factors of which the transcription repressor Snail (SNAI1), Slug (SNAI2), Twist1, and ZEB1 are the best understood. EMT can also be induced by environmental cues, such as hypoxia, which is arguably the most characterized.

As will be discussed below, while these EMT modulators function through diverse signaling pathways, all are able to induce H19/miR-675 expression. This strongly suggests that H19 /miR-675 up-regulation is a common denominator of EMT inducers, and reflects its pivotal role in this process [33].

### TGF-β induces H19/miR675 levels through the PI3K-AKT pathway

TGF-β mediates several, sometimes paradoxical, effects on cancer cells. TGF-β can function as a tumor suppressor or as an oncogene. In early stage carcinoma and in normal cells TGF-β has the ability to induce cell cycle arrest or apoptosis reflecting its tumor suppressive functions. In advanced carcinomas, TGF-β can promote carcinoma growth, invasion and subsequent metastasis. Increasing evidence shows that this paradoxical switch in TGF-β function could be attributed at least in part to the ability of TGF-β to induce and maintain EMT programs in cancer cells as well as creating an EMT-permissive microenvironment during cancer progression and metastasis.

Our recent observations show that TGF-β can induce the level of H19/miR-675 along with established EMT markers in various carcinoma models [33]. TGF-β signaling regulates the expression and activity of a variety of EMT transcription factors such as Snail and Slug. Although Slug has been shown to contribute to EMT and tumor metastasis, the molecular events leading to its induction are poorly understood. We reported recently that Slug induction by TGF-β is dependent on H19/miR-675 [33].

Several signaling pathways that induce EMT often activate the phosphatidylinositide 3-kinase (PI3K)-Akt axis. Enhanced TGF-β receptor signaling was reported to maintain hyperactive PI3K/AKT signaling, which in turn promoted EMT [34]. Furthermore, many different cancer types with elevated invasiveness, metastasis and poor prognosis are associated with hyperactivation of Akt.

Inhibiting PI3K-Akt using a specific chemical inhibitor affected EMT transcription factors as well as H19/miR-675 inductions by TGF-β. Altogether these findings indicate that cross talk between the TGF-β and PI3K-Akt signaling pathways is necessary for the induction of the EMT program. We have also provided clear evidence that Slug induction by TGF-β is H19 dependent [33].

The involvement of Ha-Ras oncoprotein and TGF-β in the induction of EMT can be studied in the well-characterized EpRas and EpRasXT mice cell model of mammary carcinogenesis and represent another opportunity to test for differential h19 expression. Ha-Ras–transformed EpH4 mammary epithelial cells give rise to oncogenic fully-polarized cells, EpRas. These cells can undergo EMT in response to TGF-β both *in vitro* and *in vivo* giving rise to the mesenchyme-like EpRasXT cells. Numerous signaling pathways have been identified to be pivotal to EMT conversion in this model including the IKK-2/IκBα/NF-κB pathway [35] and the autocrine PDGF/PDGFR loop which hyperactivates PI3K [36 ]. We have reported recently that h19 is highly expressed in mesenchymal EpRasXT cells relative to epithelial EpRas cells [33]. The functional consequences of h19 up-regulation and the signaling pathway that is responsible for h19 induction remain to be established.

On the other hand, other studies showed that TGF-β1 decreased the H19 lncRNA content of cultured fetal adrenal cells [37]. It was also reported that TGF-β1 was ineffective in promoting the H19 promoter activity in MDCK epithelial cells [38]. Thus it seems that TGF-β can modulate the expression of H19 lncRNA depending on cell type and thus can potentially induced different phenotypes. Whereas TGF-β induced H19 expression in cancer cells resulted in EMT [33], its suppressive effect on H19 expression in normal cells remain to be elucidated.

### The scattering phenotype of HGF/SF is dependent on H19 LncRNA

HGF/SF and its receptor, the tyrosine kinase MET, play a central role in many aspects of tumor progression. Their pivotal role in angiogenesis, tumor progression, and metastasis is evident from numerous studies. Furthermore, HGF/SF can induce EMT. In fact, it was the first extracellular growth factor identified as an EMT inducer [39]. Tumor cell scattering in primary tumors is the first step in metastasis, and as its name implies, HGF/SF plays a pivotal role through the paracrine loop between HGF-producing stroma and MET-expressing tumor cells. HGF/SF induces dispersion of cluster cells into single cells via an endocytosis of E-cadherin from the cell surface to the cytoplasm. In addition, HGF/SF induces scattering of epithelial cells by up-regulating the expression of Snail transcription factor which represses the expression of E-cadherin and claudin-3 genes [40].

HGF/SF was identified as a fibroblast-derived growth factor capable of inducing H19 expression and cell morphogenesis. It can also activate H19 promoter through MAP kinase or phospholipase pathways C. H19 thus may be implicated in morphogenesis and/or migration of epithelial cells in response to HGF/SF [38]. We recently presented new evidence showing that the tumorigenic and scattering effect of HGF/SF on lung cancer cell lines which express MET receptor can be attenuated by H19 knockdown. The scattering morphology of the clones induced by HGF/SF was totally dependent on H19 RNA under both normal and hypoxia recovery conditions [33].

### EMT induced multi-drug resistance is accompanied by profound H19 LncRNA induction

Sensitive and drug resistant cancer cells are two distinct groups, which differ in expression of specific genes, including several that are features of EMT [41]. Aberrant expression of the EMT transcription factors Snail and Slug alters the response to genotoxic stress. Snail and Slug promote cell survival after genotoxic stress through repressing genes involved in programmed cell death including the p53 signaling pathway [42]. Mesenchymal-like cells within a tumor are more drug resistant than epithelial-like cells [43].

Furthermore, it was proposed a long time ago that the multi-drug resistance (MDR) phenotype and cancer invasiveness are correlated. Recent mechanistic evidence revealed that EMT-inducing transcription factors increases the promoter activity and expression of the ATP-binding cassette (ABC) transporters resulting in enhanced drug resistance, migration and invasion [44].

Over-expression of H19 lncRNA has been been reported to accompany the up-regulation of a 95 kDa membrane glycoprotein (p95) observed in variants of breast and lung carcinomas that are multi-drug resistant [45]. Moreover, results show that the level of H19 RNA is elevated in the multi-drug resistant variant of hepatocellular carcinoma (HCC) cell lines. Here, the doxorubicin resistant phenotype is related to H19 over-expression [46]. Mechanistically, H19 induces P-glycoprotein expression and MDR1-associated drug resistance through the regulation of MDR1 promoter methylation.

All of these observations suggest that the EMT-drug resistance phenotype may involve H19 lncRNA. To test for such an association, we recently utilized the well-characterized cisplatinum resistant ovarian adenocarcinoma model (A2780cis), which has both the morphological and the phenotypic hallmarks of EMT and represents a good model to test for differential expression of H19 RNA compared to its epithelial parental line (A2780). H19 RNA was largely induced in the mesenchymal cisplatinum resistant variant along with EMT markers including Slug transcription factor [33].

We propose that H19 lncRNA contributes to the multi-drug resistant phenotype through various distinct mechanisms. Through an epigenetic mechanism, H19 RNA regulates MDR1 promoter methylation leading to its induction. Furthermore, through the up-regulation of Slug EMT transcription factor, H19 may have a dual function in suppressing genes involved in multi-drug resistance while inducing genes related to the EMT phenotype. We are currently in the process of validating this issue experimentally. The possible contribution of miR-675 to this process may represent an additional scenario whereby gene products involved in MDR and EMT phenotypes are targeted.

### Hypoxic cue: H19 and EMT

Hypoxia is created in a tumor mass when oxygen consumption is greater than oxygen supply. Hypoxia, which is the most common feature of solid tumors, is a major environmental cue that can trigger and maintain the EMT program as an adaptive response by tumor cells, resulting in enhanced metastatic potential. Overall survival rates of cancer patients are inversely correlated to the level of hypoxia in their tumors. Hypoxia not only induces EMT, thus contributing to an increased risk of metastasis, but is also involved in the resistance against conventional cancer therapies.

We were the first to report that hypoxia represents a major environmental cue for the induction of the H19 RNA. Recently it was shown that miR-675 is also hypoxia responsive. We investigated the molecular mechanism by which hypoxia induces H19 expression. We found that interplay between two signaling pathways determines the up-regulation of the H19 RNA upon hypoxia, whereby the HIF1α signaling pathway induces H19 while the p53 signaling pathway inhibits H19 induction [47]. It is well established that both signaling pathways are essential in the adaptation of cancer cells to hypoxic stress. They can directly or indirectly interact in either a synergistic or an antagonist manner on certain important cellular pathways to modulate the fate of hypoxic cancer cells [48]. Hypoxia selects for cancer cells having a decreased apoptotic phenotype and increased metastatic potential [49, 50]. This was explained by the loss of wild type p53 function and/or due to the selection of cells expressing functional mutant p53 [51]. It is known that hypoxia can induce p53-dependent apoptosis resulting in the hypoxia-induced selection of mutant p53. This process can therefore facilitate the clonal expansion of cancer cells with compromised p53 function [51].

The H19 lncRNA is largely induced within this context. We showed that hypoxic cancer cells with wild type p53 cannot induce H19 lncRNA [47]. Hypoxic cancer cells with null p53 or different p53 mutants induce H19 lncRNA to varying degrees, even by as much as 100-fold or more. In order to substantially induce H19 lncRNA in hypoxic cancer cells with wild type p53 function, both inhibition of p53 and overexpression of HIF-1α are needed [47].

By applying whole genome expression profiling, we showed that H19 knockdown modulates the expression of genes involved in angiogenesis, survival and tumorigenesis in hypoxic stress. We further analyzed the functional consequences and showed when cells that are devoid of H19 expression undergo hypoxic stress, they fail to form colonies in soft agar after hypoxia recovery whereas cells that possess H19 can form colonies. Furthermore, we showed that H19 RNA confers a proliferative advantage under hypoxic stress [52].

Genes that regulate angiogenesis and blood vessel development are among those modulated by H19 knockdown [52]. We provided further support for these findings by showing that xenografts of tumors derived from H19 over-expressing bladder cancer cells were more vascularized relative to the control. Other H19-modulated genes are implicated in the survival/apoptotic decision in hypoxic stress, while others are implicated in tumor progression migration, invasion and metastasis [52, 53].

Hypoxia also is a major cue for tumor cell motility and invasion as it triggers EMT through HIF1α, a potent activator of EMT transcription factors. Proteolytic activity at the invasive front is enhanced by hypoxia for example through up-regulation of urokinase-type plasminogen activator receptor, thus stimulating cell motility by altering integrin/ECM interactions. Furthermore E-cadherin expression is repressed by hypoxia thus facilitating disruption of tissue integrity. Hypoxia also suppresses anoikis, a type of the apoptotic cell death due to the lack of adhesion to ECM [54], and enhances angiogenesis and lymphogenesis (For review see [55]).

We recently used the MDA-MB-468 breast cancer cell line, which expresses a mutant form of p53 and undergoes EMT when cultured in hypoxic conditions [56], to study H19 and miR-675 expression together with the EMT markers. Interestingly MDA-MB-468 cells cultured in hypoxic conditions demonstrated an approximately 100-fold increase in H19 mRNA compared to cells that were maintained in normoxic conditions. The EMT transcription factors Snail and Slug were induced, as was miR-675 [33]. The molecular mechanism by which H19 RNA enhances hypoxic EMT remains to be determined.

**Figure 1:**
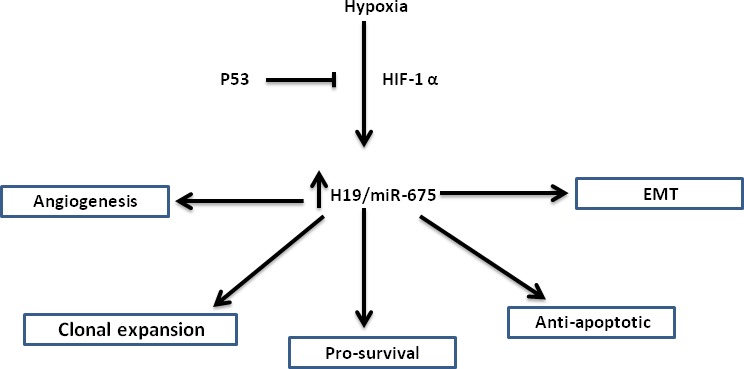
The various roles of H19/miR-675 in the hypoxic stress response Hypoxic induction of H19/miR-675 is p53 and HIF1-α dependent. Shown are different phenotypes in which H19/miR-675 is involved, placing this axis at a pivotal place in hypoxic stress accommodation, largely regarded as the most dangerous step in cancer progression.

To sum up, H19 lncRNA has multiple functions in hypoxic selection and adaptation. H19 increases short- and long-term survival by up-regulating survival genes and inhibiting apoptotic genes, and increases long-term survival by the induction of angiogenesis. H19 contributes to clonal expansion by enhancing proliferation under hypoxic stress of p53 mutant cells. H19 is also involved in the EMT program during hypoxic stress. The various roles of H19 lncRNA in hypoxic stress are summarized in (Figure 1).

## MECHANISTIC INSIGHTS INTO THE ROLE OF H19 IN EMT: MULTIPLE ROUTES TO ONE TARGET

**Figure 2:**
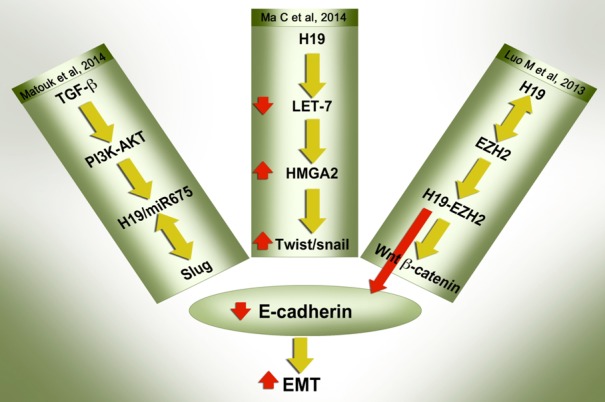
H19/miR-675 suppress E-cadherin to induce EMT through multiple modes of action E-cadherin suppression, which is universally regarded as the hallmark of the EMT process, is mediated by H19/miR-675 through different mechanisms as reported recently. The detailed description of the pathways are described in the text. Whichever signaling pathway is involved in the up-regulation of H19/miR-675, or by whichever mechanism H19 modulates its downstream targets, the end result is one phenotype - the ablation of E-cadherin.

EMT inducers are involved in several different signaling pathways. As discussed above, regardless of which signaling pathway is activated, all ultimately induce H19/miR-675 together with EMT markers, thus reflecting the pivotal role of H19 in this process. Interestingly, whichever signaling pathway is involved in the up-regulation of H19/miR-675, or by whatever mechanism H19 modulates its downstream targets, the end result is a single phenotype, that in which E-cadherin is ablated (Figure 2). The functional translation of this important molecular event is the ability of H19 /miR-675 to enhance cancer cell invasion *in vitro* and metastasis *in vivo*. Next we review the diverse signaling pathways through which H19/miR-675 can ablate the expression of E-cadherin in the context of EMT.

### The H19/miR-675/Slug/E-cadherin axis

Since EMT transcription factors are known to transcriptionally repress the E-cadherin protein, the possibility that H19 lncRNA can induce these EMT transcription factors is an attractive option to explore. Indeed, we showed that Slug induction triggered by TGF-β is largely dependent on H19 RNA as discussed above [33].

In a pancreatic cancer cell model, H19 over-expression alone, without triggering by EMT inducers, was sufficient to cause a profound induction of Slug, but not Snail expression, with subsequent ablation of E-cadherin protein level [33].

As H19 is a miRNA precursor [7], it could at least in part function through miR-675. Where miR-675 was analyzed, we noticed a harmonic up-regulation with H19 induction. Numerous miR-675 targets have been identified including those that can affect the proliferation/differentiation decision and we recently showed for the first time that miR-675 can also induce EMT [33].

H19 up-regulates Slug expression through a mechanism that involves miR-675. This is accompanied by suppression of the Slug target E-cadherin [33]. However the identity of the miR-675 target(s) that caused the up-regulation of Slug remains to be elucidated. H19 and miR-675 are up-regulated in many types of cancers and in response to the same EMT trigger. It remains unclear how this is accomplished, taking into consideration that miR-675 is processed at the expense of H19 lncRNA. One option is that miR-675 could have its own regulatory sequence, besides being processed from H19. This has yet to be investigated.

Since it is a transcription factor, we hypothesized that Slug could up-regulate H19 expression by enhancing its transcription thus generating a positive feedback loop. Through this loop Slug could boost its own level by transcriptional activation of H19 lncRNA thus generating excessive miR-675 to inhibit Slug inhibitor. Using an ovarian cancer model (A2780), we showed that Slug induces H19 expression and activates its promoter [33]. This positive feedback loop could be responsible for inducing and then maintaining the mesenchymal phenotype of tumor cells.

In accordance with these results, it was recently shown that H19 promotes glioma cell invasion through miR-675. H19 expression and miR-675 level were closely correlated with tumor grade in different glioma data sets. MiR-675 modulated Cadherin 13 expression by directly targeting the binding site within the 3′ UTR [57]. Current studies have highlighted the role of CDH13 as a tumor suppressor in numerous cancer types [58].

### H19/Let-7 /Hmga2 axis

The high mobility group protein A2 (HMGA2) is a non-histone chromatin binding factor involved in many biological processes including cell growth and differentiation with the ability to induce a mesenchymal phenotype characterized by a strong down-regulation of E-cadherin [59]. Physical interaction between HMGA2 and the Smads, leading to increased binding of Smad proteins on the *SNAIL1* promoter thus activating it to induce EMT in response to TGF-β has been reported [60]. Furthermore Smad signaling also induces HMGA2 levels upon TGF-β stimulation which is an example of a feed-forward mechanism by which Smad could regulate many of its targets [59]. Furthermore, it was reported that HMGA2 maintains oncogenic Ras-induced EMT in pancreatic cancer cells [61].

HMGA2 gene has a long 3′UTR that can be targeted by, among others, let-7 tumor suppressor microRNA [62, 63]. Research indicates that a major mechanism of oncogenic Hmga2 translocations associated with various human tumors is the loss of let-7 repression [62]. Recently it was shown that H19 RNA can function as a molecular sponge for let-7 tumor suppressor miRNA, as it harbors both canonical and non-canonical binding sites for the let-7 family of miRNAs [64]. H19 promotes tumor cell migration and invasion by sponging let-7, thus relieving the inhibitory effect of let-7 on its targets, resulting in an increase of metastasis-promoting genes including Hmga2, c-Myc and Igf2bp3 [65]. Knockdown of H19 resulted in concomitant decrease of EMT transcription factors Twist and Snail and EMT cytoskeletal marker Vimentin and an increase of the expression of epithelial cell surface marker E-cadherin. In this particular study it was also shown that the anti-diabetic drug metformin, which is known to have anti-cancer activity, can inhibit tumor cell migration and invasion partly by down-regulating H19 epigenetically by DNA methylation of its promoter [65].

Further support for the role of the H19/let-7/Hmg2 axis in the promotion of tumor metastasis was recently provided in a study of H19 in pancreatic ductal adenocarcinoma (PDAC) [14]. H19 expression was overexpressed in PDAC cells compared to adjacent normal cells with a higher levels of up-regulation in primary pancreatic tumors which subsequently metastasized, compared to those that did not metastasize. PDAC is among the most lethal cancer types with an unprecedented ability to disseminate and metastasize. Knockdown of H19 impaired PDAC invasion and the metastasis phenotype could at least in part be explained by the ability of H19 to sponge let-7 thus relieving its inhibitory effect on HMGA2-mediated EMT [14].

Altogether, this represents a previously unknown mechanism whereby a lncRNA acts as a molecular sponge for tumor suppressor microRNA resulting in up-regulation of its target oncogenes. Further validated patterns through which H19 lncRNA can perform its function can be found in a recent review [66].

### Epigenetic H19/EZH2/Wnt/β-catenin/E-cadherin axis

EZH2 (enhancer of zeste homolog 2 (Drosophila)) is a member of the polycomb-group (PcG) protein family that functions as a histone H3 Lys 27 (H3K27) methyltransferase when it is part of the polycomb-repressive complex 2 (PRC2). Both HOTAIR and XIST have been reported to interact with EZH2 to transcriptionally repress chromatin thus regulating gene expression epigenetically [67].

EZH2 has been shown to mediate E-cadherin repression so promoting metastasis of oral tongue squamous cell carcinoma [68]. Furthermore, EZH2 promotes tumor cell migration and invasion via epigenetic repression of E-cadherin in renal cell carcinoma [69]. Sox4, a master regulator of EMT, directly regulates the expression of *Ezh2* to induce EMT in response to TGF-β [70]. Ezh2 is able to functionally replace Sox4 during EMT and regulates the expression of a number of EMT-associated genes. EZH2 can induce EMT of prostate cancer cells by down-regulation of DAB2IP, a tumor-suppressive Ras GTPase-activating protein (RasGAP) [71]. Thus EZH2 is a master epigenetic modifier that is involved in the metastatic cascade and its expression correlates with metastasis-free survival [72].

The association of H19 lncRNA with the epigenetic machinery is a newly described phenomenon by which H19 lncRNA can perform its function. Recently it was reported that methyl-CpG-binding domain protein 1 (MBD1) is a physical and functional partner of H19 lncRNA that is required for the regional control of five other genes located on the same imprinted cluster on chromosome 11 [73].

In the context of metastasis, the expression of H19 lncRNA is markedly increased in biopsies taken from primary bladder cancer that subsequently metastasized. H19 regulates bladder cancer metastasis through its association with EZH2[15]. This association resulted in increased Wnt/β-catenin activation which is tightly linked to EMT. Interestingly, it was shown that association of H19 with EZH2 can resulted in the suppression of E-cadherin through direct and indirect mechanisms. H19 can directly suppress E-cadherin promoter activity, and also indirectly suppress it by activation of the Wnt signaling pathway; either way requires EZH2 [15].

## THE ROLE OF H19 IN MET

While there are conflicting reports on the role of H19/miR-675 in tumorigenesis [52, 53, 74-76], the prevailing view is that H19/miR-675 behaves like an oncogene. Research into the role of H19/miR-675 in tumor metastasis has similarly lead to contradictory findings.

It was reported that H19 can epigenetically activate the miR-200 pathway by increasing histone acetylation. The miR-200 family has been found to inhibit EMT and cancer cell migration. Thus it was deduced that H19 contributes to MET and to the suppression of tumor metastasis [77]. High and low miR-200 expression levels are well correlated with the epithelial and mesenchymal phenotypes respectively [78, 79]. Furthermore cancer stem cell traits are associated with decreased miR-200 and increased expression of EMT transcription factors [80].

Recently, it was shown that miR-675 mediates down-regulation of Twist1 and retinoblastoma in alpha fetoprotein-secreting hepatocellular carcinoma. Overexpression of miR-675 alters cellular morphology, reduces invasive potential and increases anchorage-independent growth capacity. These findings indicate a mesenchymal-to-epithelial transition, associated with a reduction in the Twist1, a key EMT mediator [81].

At the first glance the role of H19/miR-675 in metastatic process seems controversial. However EMT as well as MET are recognized as a critical events for metastasis of carcinomas. MET at sites of metastases has been postulated to be a central part of the process of metastatic tumor formation (for review see [82]). While EMT is critical for the initial transformation from benign to invasive carcinoma, MET is critical for the colonization and growth in later stages of metastasis. The epithelial phenotype seems to be central for secondary tumor formation. This was clearly shown in isogenic mouse breast cancer cell lines (67NR, 168FARN, 4TO7, and 4T1) reflecting the multistep model of metastasis. Only 4T1, which acquired epithelial properties, formed macroscopic lung and liver metastases, indicating the importance of the epithelial properties [83].

Utilizing the same model, we recently showed that h19 gene is differentially expressed. Both 168FARN, which forms regional lymph node metastasis, and 4TO7, which metastasizes to the lung but does not colonize, had moderate up-regulation of h19 RNA when compared to non-metastatic 67NR. Interestingly, the highest expression level was detected in 4T1 which may reflect the importance of h19 not only in the initial EMT events but also for the final MET events as well. Furthermore, cells over-expressing H19 can colonize the lung faster and establish much larger tumor foci compared to control [33].

Furthermore, and most importantly, we consistently detected high H19 expression levels in the invasive front of primary tumor biopsies (reflecting EMT). H19 RNA was also highly expressed in all common metastatic sites examined regardless of the primary origin (reflecting MET) indicating that H19 activation is a general phenomenon of the metastatic events [33].

In conclusion, the pro-metastatic and anti-metastatic roles attributed to H19/miR-675 in different models may reflect the different roles of this axis in both the EMT and MET events and thus are complimentary. The molecular factor(s) and/or environmental cues that dictate and govern the role of H19/miR-675 in both critical events remain to be elucidated.

## THE H19/MIR-675 AXIS ORCHESTRATES EMT/MET PHENOTYPES THROUGH MODULATING SLUG-MIR-200 LEVELS - A WORKING HYPOTHESIS

**Figure 3:**
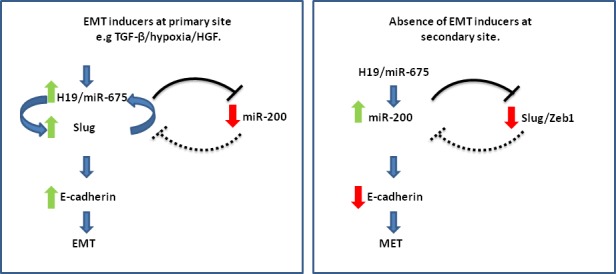
The H19/miR-675 axis orchestrates EMT/MET phenotypes by modulating Slug-miR-200 levels: A working hypothesis H19/miR-675 either activates the expression levels of Slug, which is present in a positive feedback loop with H19 in the context of EMT in the invasive front of the primary tumor (left), or activates miR-200 at the secondary site to promote colonization and differentiation and subsequent induction of MET (right). These orchestrated events are tightly regulated through a delicate interplay between both environmental niche signals which establish the H19-miR675-Slug positive feedback loop at the primary site, and a complex network of signaling pathways or intrinsic factors like P53, which can favor the loop formation (left). EMT inducing niche signals are absent at the secondary site and this breaks the loop between Slug and H19, and can cause reversible epigenetic modifications of relevant genes at the molecular level that disfavor the positive loop. The H19/miR-675 axis in this novel niche of secondary growth would modulate factors facilitating the up-regulation of the miR-200 axis and thus induce differentiation at the secondary site (right).

In this section, we propose a working hypothesis to deal with various facets of the role of H19/miR-675 in the metastatic process and illustrated in (Figure 3). We have attempted to combine seemingly conflicting data from different reports under one proposed general scheme underlying the different roles of H19/miR-675 in the metastatic cascade.

We assume that the critical point is the ability of H19/miR-675 to activate the expression levels of either Slug in the context of EMT or miR-200 in the context of MET, and consequently H19/miR-675 could be regarded as a determinant orchestrator of the expression levels of the two factors [Figure 3]. As discussed above, H19/miR-675 can up-regulate both of these factors under different conditions [33, 77]. It is interesting that a mutual suppressive relationship is known to exist between these two pivotal EMT/MET modulators. For instance, in a model of prostate adenocarcinoma that harbors PTEN and TP53-null mutations and progresses to EMT via TGF-β, it was shown that Slug is a direct repressor of miR-200 transcription [84]. Furthermore it was shown that there is a mutually inhibitory feedback circuit for miR-1 and miR-200 with Slug. Slug is also a direct target for miR-200. Slug depletion inhibits EMT and prevents subsequent induction of Snail and Zeb1/2 in this model [84].

We propose that the role of H19/miR-675 here is to shift the balance in favor of either Slug or miR-200. H19/miR-675 axis would shift the balance towards Slug in the invasive front of the primary sites and in circulation resulting in subsequent suppression of E-cadherin and induction of EMT. On the other hand, H19/miR-675 would shift the balance towards miR-200 at the secondary site to promote colonization and differentiation and subsequent induction of MET. These orchestrated events are tightly regulated through a delicate interplay between environmental signals (extrinsic factors), and a complex network of signaling pathways (intrinsic factors). Both extrinsic and intrinsic determinants are essential modulators for the EMT/MET phenotype, and should also modulate the H19/miR-675 axis accordingly [33].

We suggest that the positive feedback loop between H19/miR-675 and Slug discussed above may have a critical role in the EMT/MET decision. This loop could be established, maintained and regulated in response to numerous environmental signals that are present in the primary tumor niche, such as TGF-β, HGF, and hypoxia [33]. The loop could also be strengthened by the intrinsic factors that pre-dispose the cell to undergo EMT in response to these signals, for example P53 (epi) mutations, HMGH2, let-7, active Wnt signaling, all reported to modulate or be modulated by the H19/miR-675 axis [14, 15, 47, 64, 65].

Environmental signals that induce EMT are not only present at tumor primary sites but also accompany the tumor cells all the way from the primary site to the blood circulation, but cease at the secondary sites. For example, it was reported that circulating tumor cells are provided with EMT inducing signals through platelets [4]. We propose that this may maintain and boost the H19/miR-675-Slug positive loop through the circulation. This assumption may be supported by our observation that circulating tumor cells have very high levels of H19/miR-675 (unpublished data). In fact it was shown in this study that TGF-β is produced by the platelets [4].

When the tumor cells settle at the secondary site, the EMT inducing environmental signals will be absent and this will weaken and break the loop between Slug and H19, and thus contribute to the reversion to MET and the up-regulation of E-cadherin. The H19/miR-675 axis in this novel niche of secondary growth up-regulate miR-200. Epigenetically modulated factors in this new environment may contribute and facilitate the up-regulation of miR-200 axis.

It was postulated that for colonization and macro-metastasis to occur, MET and re-differentiation are mostly triggered by the changing environmental niche of the secondary tumors that can cause reversible epigenetic modifications of relevant genes at the molecular level [85]. A high proportion of carcinomas of diverse origins differentiate in distant metastatic sites to an even greater degree than the corresponding primary sites. There is a heterogeneous subpopulation of cells with various degrees of differentiation at the sites of metastasis. Taking into consideration the known role of miR-200 in inducing differentiation at secondary sites [85], and the role of H19 in inducing miR-200 in the context of MET [77], then it is feasible to suggest that H19 is involved in differentiation at secondary sites possibly at least in part via up regulating miR-200.

Intrinsic factors are also important determinants in the EMT/MET decision via the H19/miR-675 axis. For instance, loss of TP53 through genetic or epigenetic mutations or through silencing would result in elevation of Slug expression [86]. Furthermore, we and others have reported that wild type p53 (p53^wt^) is a suppressor of H19 [47, 87]. Thus it is reasonable to suggest that loss of p53 through epi(mutation) will foster the positive loop between H19 and Slug triggered by extrinsic factors towards full EMT conversion. This could be accomplished via several routes. Hypoxic EMT induction of H19/miR-675 and subsequent induction of Slug is expected to be p53 dependent [33]. Furthermore since it was reported that miR-200 is suppressed by Slug [84] and induced by p53^wt^ [88], so would be expected to have low expression levels in the context of P53 epi(mutation), further boosting the EMT phenotype.

In support of this suggestion, it is very interesting to note that the function of H19 as a MET inducer was reported by two separate investigators in a model of hepatocellular carcinoma cell lines with a p53^wt^ background and in the absence of an EMT inducer. In the first study, H19 was found to epigenetically up-regulate miR-200 and contribute to MET in two cell line models (SMMC7721 and HCCLM3), both possessing p53^wt^ [77]. In the second study, overexpression of miR-675 altered cellular morphology, reduced invasive potential, and increased anchorage-independent growth capacity. These findings are consistent with a mesenchymal-to-epithelial transition, associated with a reduction in the expression of the key EMT mediator, Twist1. HepG2 and Sk-Hep-1 cell lines were used in this study also with p53^wt^ background [81].

The seemingly controversial roles of H19/miR-675 axis in metastasis process may also help to explain the controversial roles of H19/miR-675 axis in tumorigenesis in general. We propose that that H19/miR-675 axis may function as a tumor suppressor or as an oncogene by at least in part through modulating the differentiation process. In this aspect H19/miR-675 may impose its tumor suppressive role through acting as a pro-differentiating factor or may function as an oncogene by promoting stemness/de-differentiating process. Both roles of H19/miR-675 axis have compelling supports in the literature [64, 89-92]. However it remains to be determined whether the same molecular pathways described above for the role of H19/miR-675 axis in orchestrating the EMT/MET, also mediate the pro and anti tumorigenic function of H19/miR-675 axis.

## OUTSIDE CANCER: ASSOCIATION OF H19 WITH PHYSIOLOGICAL EMT PROCESSES

Numerous physiological processes involving cellular invasion, blastocyst implantation, placental development, tissue repair and remodeling, wound healing, and loss of pluripotency, all involve EMT. Interestingly, all these physiological processes are associated with H19 activation (for review see [93]).

Similar to metastatic tumour cells, trophoblast giant cells (TGC) breach basement membranes and invade deeply into maternal decidua. Their angiogenic properties coupled with tissue remodeling enable them to redirect maternal blood flow towards the implantation site. Recently it was reported that one of the most abundant non-coding RNAs found in the TGC RNA-seq data set was H19. The authors proposed that H19 plays a role in controlling the extent of invasiveness of the TGC cell population [94].

We have also reported that H19 is highly expressed in human placental extravillous cytotrophoblast cells. These are polyploid cells that invade the uterus [95]. H19 is also highly activated at the time of implantation in the developing murine embryo [96].

Aberrant EMT also elicits disease development in humans, including rheumatoid arthritis (RA) and fibrosis. Studies indicate that the PI3K/AKT/HIF-1α pathway is associated with hypoxia-induced EMT in fibroblast-like synoviocytes of RA [97]. We have shown as discussed above that these pathways also resulted in the up-regulation of H19 in the context of EMT response to both TGF-β and hypoxia [33]. Interestingly, RA is a known non-cancerous pathological process that is associated with activation of H19 RNA [98]. We propose that activation of H19 by either oncogenic signals or non-cancerous pathological signals converge at the same signaling pathway that activates H19 and its downstream targets. It is tightly regulated with transient nature in physiological conditions while the reverse is true for cancer and pathological situations [99].

## CLINICAL DATA: SUPPORT THE INVOLVEMENT OF H19 IN METASTATIC PROCESS

### H19 expression in liver metastasis from a range of human carcinomas

The liver is one of the major sites of cancer metastasis especially for carcinomas originating in the gastrointestinal tract, breast, and lung. We investigated H19 expression in hepatic metastases of representative paraffin wax blocks of metastatic carcinoma originating from colorectal, pancreatic, ovarian, breast and gastric cancer. These blocks were gathered from the pool of routinely prepared blocks from core and wedge liver biopsies and partial hepatectomy specimens taken from 80 patients with hepatic metastases. Using non-radioactive ISH technique, we found that H19 expression was detected in a high percentage of tumor biopsies with a very high expression in more than 50% of the biopsies. There was also a strong staining in the adjacent liver parenchyma in some of the samples.

In many of the metastases there was a significant desmoplastic reaction, which mostly showed similar staining to the adjacent metastatic carcinoma [100]. This is not surprising because it is known that H19 is expressed within both epithelial and stromal components of invasive adenocarcinomas [101]. The staining of the desmoplastic reaction might also be related to the alleged contribution of H19 to epithelial–mesenchymal interactions. Thus targeting the tumor microenvironment expressing H19 RNA may render the treatment option more effective, as will be discussed below.

Furthermore, the growth of liver metastases after hepatectomy and its correlation with levels of H19 and HGF RNAs were evaluated by our group on an orthotopic model of rat colon liver metastases. HGF and H19 RNAs levels in tumors were substantially elevated after hepatectomy. There was a direct positive correlation between the level of H19 RNA and the extent of liver resection. We proposed that HGF has an important role in overexpression of H19 resulting in a rapid growth of metastases in the remaining liver after hepatectomy [102].

In summary, the expression of H19 can be detected in liver metastasis whatever the primary tumor, with variable levels of expression from patient to patient.

### Lung metastasis and colonization

A series of *in vitro* and *in vivo* studies using animal models indicated that H19 is involved in lung metastasis and colonization. High H19 expression in lung metastasis has also been detected in a limited numbers of human biopsies irrespective of tumor primary origin [33]. Larger numbers of biopsies are needed to firmly establish the prognostic values of H19 expression in lung metastasis.

Other studies indicate that H19 RNA is among the most increased RNAs in the PANC pancreatic cancer cell line that forms lung metastasis as compared to the parental cells. The cell proliferation rate was not altered in the H19 siRNA transfected metastatic cells, but cell migration was significantly decreased in the cells. These findings suggest that H19 lncRNA is important for the metastasis and cancer stem cell functions of pancreatic cancer [103].

In order to establish the mechanistic role of H19 in the metastatic cascade *in vivo*, we recently showed that H19 RNA greatly enhances lung colonization. Forced expression of H19 RNA in H358 cells enhanced their capacity to generate lung metastases when injected intravenously into the tails of athymic mice [33].

### Brain metastasis

We have shown that H19 is highly expressed in high grade gliomas. Furthermore, significant H19 expression in brain metastases was also detected [104]. In a larger study, it was found that H19/miR-675 expression correlates with glioma grade. H19/miR675 expression was significant higher in high grade glioma tissues than in low grade ones. It was shown that the miR-675 enhances the invasion in glioma cells by directly targeting CDH13 [57].

## TOWARDS TRANSLATIONAL MEDICINE UTILIZING THE H19/MIR675 AXIS

Preventing metastasis formation and targeting existing metastasis is currently an unmet clinical need. Metastasis is responsible for the vast majority of the catastrophic outcomes of cancer. Palliative medicine in most cases is almost the only option available currently. Understanding the complex biology that underlies metastasis should lead to the development of novel therapeutic strategies.

H19 lncRNA is at the core of multiple signaling pathways that dictate a tumor's deadly signature. The EMT-MET pathways are of extremely great therapeutic interest and are a major clinical challenge in cancer treatment. Besides being responsible for cancer spreading and colonization, EMT also contributes to therapeutic drug failure, and the generation of cancer cells with stem-like properties. In light of the growing body of evidence showing H19/miR-675's involvement in the two pathways, this axis should stand as a first priority target for cancer therapy.

Various strategies for targeting the EMT pathway have been proposed. Blocking micro-environmental EMT inducers and their receptors on cancer cells, and blocking signal transducers and EMT transcription factors and internal positive loops, would potentially benefit cancer patients with a high risk of metastasis and could be used as an adjuvant therapy that also will have the potential to increase susceptibility to chemotherapy. Targeting the mesenchymal phenotypes, and finally, targeting MET conversion and colonization, which are critical events in metastatic cascade, should constitute very attractive broad strategic approaches, but unfortunately every approach has its own limitations (for review see [105]).

Taking into consideration the different roles of H19/miR-675 in the metastatic cascade, the wide micro-environmental factors that induce H19/miR-675 in the context of EMT, and the diverse EMT signaling pathways activating it, it would be reasonable to assume that targeting cells with H19/miR-675 expression would be a very attractive way to attempt the aforementioned approaches.

One way to specifically target H19 expressing cells is through transcriptional targeting. During recent years we have developed a DNA-based drug (a plasmid vector) constructed from the H19 regulatory sequence (H19 promoter) that is used to drive the expression of Diphtheria toxin A chain (DT-A) specifically to the cancer cells that express the H19 lncRNA. Hereafter the therapeutic plasmid will be referred to as BC-819. The DT-A chain is a strong inhibitor of protein synthesis that catalyzes the ADP-ribosylation of diphtamide, a post-translationally modified histidine residue present in the EF-2 where it catalyzes the transfer of ADP-ribose from nicotinamide adenine dinucleotide to EF-2, halting protein synthesis and killing the cell.

The cell-specific expression of the DT-A chain driven by the H19 promoter region was shown to kill cancer cells in a variety of targeted indications, i.e. bladder, pancreatic, and ovarian cancers, as well as cancers metastatic to the liver. These non-clinical proof-of-concept studies support a potential clinical efficacy for BC-819 in patients whose tumor cells express H19 RNA. Indeed BC-819 has been administered to patients with TCC of the bladder in three clinical trials (Phase 1/2a: 18, Phase 2b: 47, Phase 1 combination trial: 18) and to four patients during a named patient compassionate use program. A total of 87 patients have therefore been treated with BC-819/PEI by intravesical route [106]. A total of 24 patients with pancreatic cancer have been treated with intratumoral BC-819. Furthermore a total of 15 ovarian cancer patients have been treated with intraperitoneal BC-819 [−107 and manuscript under preparation]. In addition, two patients with hepatic metastases were treated with BC-819 by intratumoral and hepatic artery infusion, respectively. All of these clinical studies indicate an excellent safety profile of BC-819 and good efficacy.

Pre-clinically, we have checked the utility of BC-819 as anti-metastatic drug using several animal models. In an orthotopic rat model of metastatic colon carcinoma, two injections separated by four days of 50 μg BC-819 as naked DNA or complexed with PEI were able to inhibit the growth of CC531 rat colon adenocarcinoma cells implanted into the subcapsular space of rat livers [108, 109]. The therapeutic potential of BC-819 delivered through the hepatic artery was also tested in this model. All of the tumor nodules treated with the control vector increased in size, while tumor nodules treated with a range of concentrations of BC-819 shrank in size in more than a third of the treated rats.

The orthotopic rat model of metastatic colon carcinoma described above was further used as the animal model for hepatectomy of liver metastases. Partial hepatectomy is the best treatment option for liver metastases. However, the overall recurrence rate is high and may reach 60% [110]. The progression of tumors and metastatic growth after liver resection has been noted in experimental and clinical studies. This progression has been related to the increase in hepatocyte growth factor (HGF) level after hepatectomy. As discussed above, H19 is a target gene for HGF. Thus, it was of interest to evaluate the impact of partial hepatectomy on tumor growth as well as to evaluate the correlation between H19 and HGF levels in tumors and in the liver after hepatectomy in the animal models being used to evaluated BC-819 therapy of liver metastases [102].

Tumor size increased 2-fold in rats that underwent 70% hepatectomy compared to sham operated animals with an increase of both HGF and H19 RNAs. The increase in HGF level after partial hepatectomy results in rapid hepatocyte proliferation, and also causes the overexpression of H19 in the liver tissue and tumor, which explains rapid growth of liver metastases after partial liver resection. This fact has very important clinical implications and should always be taken into consideration before hepatectomy, since partial hepatectomy with curative intent may result in rapid growth of occult liver metastases in the remaining liver and, may also influence extrahepatic tumors.

In one of our studies we used a hamster orthotopic pancreatic carcinoma model to evaluate the possible advantages of local treatment of pancreatic cancer with BC-819 in combination with systemic administration of gemcitabine, the standard of care in pancreatic cancer therapy. This study demonstrated that BC-819 used in combination with gemcitabine is more efficient than gemcitabine alone at controlling the tumor growth progression (reflected by lower tumor volume) and at preventing the occurrence of metastases [111].

## SUMMARY AND FUTURE PERSPECTIVES

Studies over the last decade have revealed that the H19 lncRNA is a master player in tumor biology. H19 RNA is frequently deregulated in almost all tumor types tested, and contributes to both cancer initiation and progression. Furthermore preclinical and clinical studies have highlighted its therapeutic potential.

In recent years there have been many reports in the literature on the breadth of H19 inducers in the context of EMT. EMT triggered by multiple extracellular cues through diverse signaling pathways seems to have a general feature - the ability to induce H19/miR-675 expression. Not surprisingly, H19/miR-675 modulates one of the major gene products of the EMT process, in that it suppresses E-cadherin. Loss of E-cadherin is universally regarded as the hallmark of the EMT process.

Despite enormous breakthroughs in our understanding of the role of H19/miR-675 in tumor progression, many important questions still remain to be answered. Although the role(s) of H19 in the EMT process is/are mediated at least in part by miR-675, H19 itself can act independent of miR-675, for example as a sponge for let-7. It is not clear whether H19 and miR-675 have complementary roles or cancer type-specific activation modes, or if the activation of either one can lead to different phenotypic outcomes of the EMT process. Furthermore it is not clear also what triggers cancer cells to use H19 or miR-675 or both in the metastasis process.

Blockage of the H19/miR-675-Slug-E-cadherin axis appears to be an attractive strategy to disrupt the positive feedback loop between Slug and H19/miR-675. This signaling pathway that triggers and/or maintains tumor EMT could be targeted via a variety of targets that depend on the H19/miR-675 axis (transcriptional targeting by a potent toxin, H19 siRNA, antimiR-675, and so on). These approaches could potentially result in inhibiting the induction of EMT, targeting the invasive mesenchymal cell type, and locking the cells in dormancy by preventing MET. Preclinical studies are currently being performed in our laboratory to harness our current knowledge towards translational medicine.

In addition, we reported that many EMT inducers (such as TGF-β and hypoxia) can up-regulate both H19 and miR-675 in the context of EMT. We are ignorant of how this is accomplished. Taking into consideration the fact that miR-675 is processed from H19 which serves as its precursor, the prevailing view is that miR-675 is processed at the expense of H19. Since both are up-regulated in response to EMT trigger, an additional mechanism of miR-675 processing is possible. One option is that miR-675 could have its own regulatory sequences; however, this need to be investigated.

The molecular factor(s) and/or environmental cues that dictate and govern the role of H19/miR-675 in EMT and MET critical events need(s) to be elucidated.

The following questions remain to be answered:

- What is/are the targets of miR-675 that result in Slug up-regulation?
- Can Slug induce miR-675 processing.
- What are the intrinsic factors that favor the positive feedback loop between H19 and Slug in response to TGF-β.
- While P53 represents an attractive intrinsic factor candidate that determine H19/miR-675 involvement in EMT/MET decision, there is as yet no definite proof of its involvement.
- What is the exact role of H19/miR-675 in secondary site colonization? Is the H19/miR-675 axis part of the decision whether or not to differentiate at the secondary site?
- How is H19/miR-675 involved in the EMT-induced MDR process? Is this process mediated by the ability of H19/miR-675 to modulate the transcription factor that mediates EMT?
- Does the H19/miR-675 axis constitute a novel molecular mechanism for the long-standing association between invasiveness and drug resistance?
- H19 is expressed in cancerous cells but also in adjacent stromal cells. What is the role of H19 expression in stroma in the context of metastatic process?

Future research should also investigate in depth and in large scale the diagnostic and prognostic values of H19/miR-675 in cancer progression, resistance and relapse.
